# The complete mitochondrial genome of *Montipora aequituberculata* (Scleractinia, Acroporidae)

**DOI:** 10.1080/23802359.2016.1186508

**Published:** 2017-01-31

**Authors:** Yu-Min Ju, Sheng-Tai Hsiao, Fu-Wen Kuo, Jui-Hsien Wu

**Affiliations:** a National Museum of Marine Biology and Aquarium, Pingtung, Taiwan, Republic of China;; b Marine Fisheries Division, Fisheries Research Institute, Keelung, Taiwan, Republic of China;; c Eastern Marine Biology Research Center, Fisheries Research Institute, Council of Agriculture, Taitung, Taiwan, Republic of China

**Keywords:** Acroporidae, mitochondrial genome, *Montipora aequituberculata*

## Abstract

The complete mitogenome of the hexacorallia, *Montipora aequituberculata* has been amplified and sequenced. The mitogenome consists of 17,886 bp, with 13 protein-coding genes, 2 ribosomal RNA genes, 2 transfer RNA genes and a control region. It has been observed that ND5 gene is split into two parts by a large fragment of genes, which commonly presented in scleractinian coral. The overall base composition of the H-strand was A, 24.91%; G, 24.1%; C, 14.2%; and T, 36.8%, with a slight AT bias of 61.7%. The control region was 627 bp in length and located between 12S rRNA and COIII gene. Based on the neighbour-joining (NJ) tree, *M. aequituberculata* was grouped with *M. cactus*, *Anacropora matthai* and *Acropora tenuis*, and formed a clade of Acroporidae. In conclusion, the complete mitogenome of *M. aequituberculata* data may provide more informative for phylogenetic approach for corals phylogeny.

In terms of the number of scleractinian coral species, *Montipora* is a very large genus throughout the Indo-Pacific (van Oppen et al. 2004). However, *Montipora* has a taxonomic uncertainty due to confusing patterns of morphological variation, with surprising examples of convergent evolution, rapid evolution and phenotypic plasticity (Forsman et al. 2010). The mitochondrial genomes of corals have facilitated searches for more rapidly evolving mitochondrial gene regions that may resolve genus and species level (Wu et al. 2016). Although *Montipora* is one of main framework builders in marine ecologically, only one genome has been sequenced.

In this study, we report the complete mitochondrial genome of *M. aequituberculata*. The specimen was obtained from the National Museum of Marine Biology and Aquarium, Pingtung, Taiwan. The long polymerase chain reaction (PCR) and a primer-walking sequencing strategy were used to sequence the entire *M. aequituberculata* mitogenome.

The complete *M. aequituberculata* mitogenome is 17,886 bp in size (GenBank accession number KU762339), comprising 13 protein-coding genes, 2 rRNA genes, 2 tRNA genes and a control region. The ND5 gene is split into two parts by a large fragment of genes, which commonly presented in scleractinian coral, and the size of inserted fragment was usually over 10 Kb (van Oppen et al. 2000; Medina et al. 2006; Brugler & France 2007; Lin et al. 2011). All genes were encoded on the H-strand. Gene order and genome content are most similar to those of the hexacorallia (Brugler & France 2007).

The overall base composition was 36.8% T, 14.2% C, 24.9% A, and 24.1% G, with an AT content of 61.7%, which agreed with the trend of most mitochondrial genomes. The most start codons are ATG and GTG, except for ND5 (3′) that began with CCA codon. All 13 genes had complete termination codons, both TAA and TAG are used as stop codon, whereas ND5 (5′) used GGT. The control region was 627 bp in length, which was located between 12S rRNA and COIII gene, rich in AT content of 60.9%. The small and large mitochondrial ribosomal RNA genes of *M. aequituberculata* were located opposite each other on the circular genome as in other corals, unlike most other metazoans, in which mitochondrial rRNA genes are usually clustered (Boore 1999).

To determine the phylogenetic position of *M. aequituberculata*, the phylogenetic tree was reconstructed with other 21 coral species from GenBank. *Aurelia aurita* was used as outgroup for tree rooting. The neighbour-joining (NJ) molecular phylogenetic tree was constructed using 1000 bootstrap replicates (Figure 1). Result showed *M. aequituberculata*, *M. cactus*, *Anacropora matthai* and *Acropora tenuis* were grouped into a single clade of Acroporidae. This relationship has also been verified by molecular phylogenies (Lin et al. 2011; Kitano et al. 2014). In conclusion, we expect this complete mitogenome of *M. aequituberculata* can provide essential phylogenetic information of corals.

**Figure 1.**
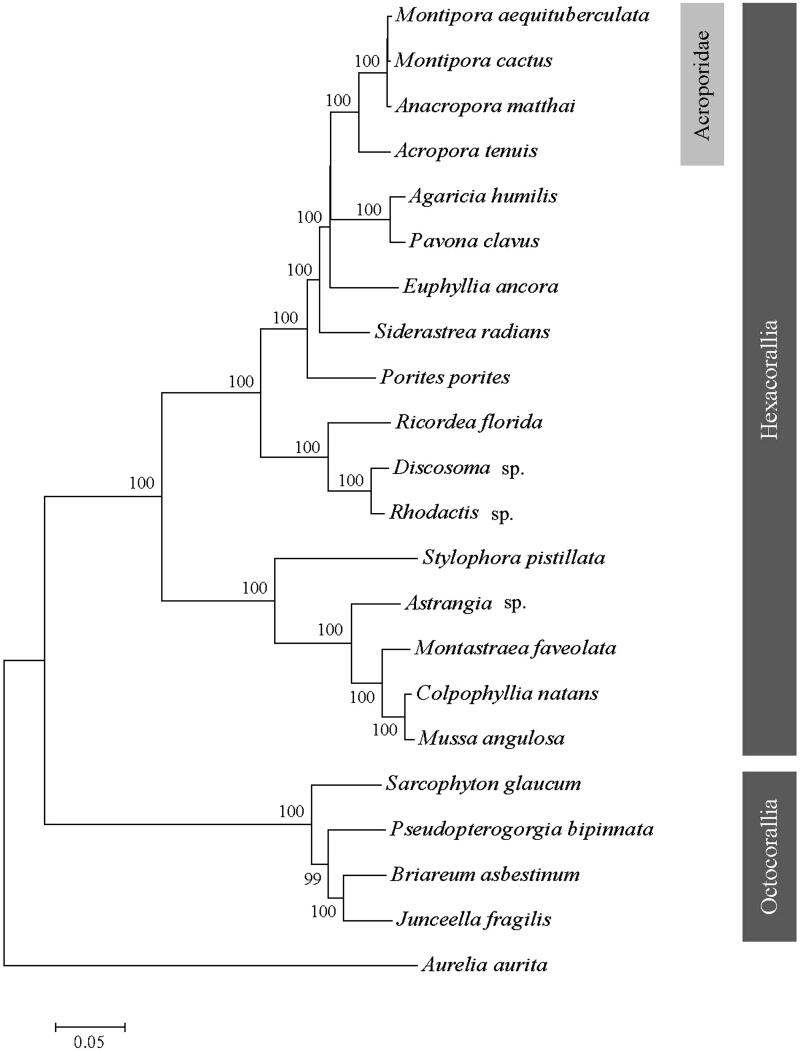
Phylogenetic tree of *Montipora aequituberculat* and other species of coral based on the complete mitogenome. Branch support values estimated by bootstrap pseudo-replicates in neighbour-joining, respectively, are shown above each branch. The Genbank accession number for tree construction is listed as follows: *Montipora cactus* (NC_006902), *Anacropora matthai* (NC_006898), *Acropora tenuis* (NC_003522), *Agaricia humilis* (NC_008160), *Povona calvus* (NC_008165), *Euphyllia ancora* (NC_015641), *Siderastrea radians* (NC_008167), *Porites porites* (NC_008166), *Rocordea florida* (NC_008159), *Dicosoma* sp. (NC_008071), *Rhodactis* sp. (NC_008158), *Stylophora pistillata* (KU762340), *Astrangia* sp. (KP072053), *Montastraea faveolata* (KP009977), *Colpphyllia natans* (JQ518289), *Mussa angulosa* (NC_008163), *Sacrophyton glaucum* (AF64823), *Pesudopterogorgia bipinnata* (NC_008157), *Briareum asbestinum* (NC_008073), *Junceella fragilis* (NC_024181), *Aurelia aurita* (NC_0088446).
